# Short- and Long-Term Effects of Maxillary Expander with Tongue Crib in Growing Open-Bite and Skeletal Class II Patients: A Retrospective Study

**DOI:** 10.3390/dj12020022

**Published:** 2024-01-24

**Authors:** Selene Barone, Francesco Bennardo, Federica Diodati, Marianna Salviati, Elena Calabria, Walter Colangeli, Alessandro Antonelli, Carmen Giudice, Amerigo Giudice

**Affiliations:** 1 School of Dentistry, Department of Health Sciences, Magna Graecia University of Catanzaro, Viale Europa, 88100 Catanzaro, Italy; selene.barone@unicz.it (S.B.); francesco.bennardo@unicz.it (F.B.); federica.diodati@studenti.unicz.it (F.D.); marianna.salviati@studenti.unicz.it (M.S.); elena.calabria@studenti.unicz.it (E.C.); colangeliw@gmail.com (W.C.); a.giudice@unicz.it (A.G.); 2 Private Practice, Via S. Rocco, 84036 Sala Consilina, Italy; carmen.giudice@libero.it

**Keywords:** anterior open bite, rapid maxillary expansion, fixed crib, jaw divergence, palatal crib, short- and long-term stability

## Abstract

The purpose of this study was to evaluate short- and long-term changes in growing patients with Class II malocclusion and open bite after rapid maxillary expansion (RME). A retrospective cohort study was conducted with 16 growing patients with open-bite malocclusion enrolled in a group treated with a rapid maxillary expander (RME) with a crib (TG), and 16 untreated patients with similar malocclusion in the control group (CG). Cephalograms were recorded before starting the treatment (T0), at the end of the latency phase (T1), and before the fixed therapy (T2) in order to analyze skeletal and dental changes in vertical, transversal, and sagittal relationships. Statistical analysis was performed with α = 0.05 as level of significance. At the end of the active expansion (T1), all subjects in the TG showed a corrected overbite with a statistically significant difference compared to the CG (*p* > 0.05). A significant decrease in jaw divergence was found in the TG compared to the CG (*p* < 0.05). At T2, all treated patients maintained a correct overbite. Statistical analysis revealed a significant decrease in maxillary, mandibular, and intermaxillary divergence in the TG compared to the CG (*p* < 0.05). This protocol could be effective in growing open-bite patients, showing a long-term decrease in facial divergence. The fixed crib allowed to normalize myofunctional activity.

## 1. Introduction

Anterior open bite (AOB) is defined by a lack of incisal contact between the upper and lower teeth. It can be considered a clinical sign of discrepancy in the vertical relationship between dental arches or skeletal structures [1,2]. Among children and adolescents, there is a prevalence of 16.52%, ranging from 25% to 38% in patients requiring orthodontic treatment, and increasing to 41.15% in patients with non-nutritive sucking habits [3]. Besides being one of the most common malocclusions, it is also one of the most difficult to treat [4]. AOB may impact aesthetic, phonetic, and psychological well-being [5]. As reported in the literature, several hereditary and environmental etiological factors are involved in anterior open bite malocclusion [5,6,7]. Extrinsic factors include non-nutritive sucking habits, abnormal tongue posture, specific dietary habits, mouth breathing (frequently associated with enlarged adenoids or tonsils), and all conditions directly related to altered orofacial growth [3,8,9]. However, genetics can also negatively influence the AOB development in subjects with a hyperdivergent skeletal growth pattern, modifying maxillary and mandibular relationships [10].

In addition to vertical changes, altered orofacial development can also include transversal discrepancies [11]. A narrow palatal vault with a unilateral or bilateral crossbite, wide buccal corridors, and a lingual inclination of the lower teeth can be recognized in association with AOB [10]. Because of the transverse deficiency, many authors have pointed out that a skeletal open bite should be treated in primary or mixed dentition to allow normal development of the anterior dentoalveolar region [12]. Several therapeutic approaches to early anterior open bite treatment have been described in the literature [13,14]. Functional (e.g., open-bite Balters’ Bionator [OBB]), fixed (e.g., quad-helix/grid), and removable (e.g., spring-loaded bite block, elastodontics) devices can be used [15]. These therapeutic methods aim to limit the vertical growth of the skeletal structures. Additionally, the palatal crib has been proposed as a treatment option for preventing thumb sucking and tongue thrusting [16].

Due to the transversal maxillary deficiency, a skeletal expansion should be performed to correct the maxillary contraction and improve the orofacial harmony [17,18]. On the other hand, behavioral control with bad habit elimination, speech therapy, and myofunctional treatment can be fundamental factors for successful therapy. The rapid maxillary expander with a crib (RME/c) exhibits orthopedic effects on maxillary development, proficiently managing oral habits for a favorable correction of anterior open bites and enhanced aesthetics [19,20]. Despite its benefits, challenges are also present, such as potential speech impairment during the adjustment period, requiring vigilant professional oversight [20]. The success of this method hinges on individual factors, emphasizing the significance of a thorough evaluation by orthodontic professionals and collaboration with speech therapists.

Several studies reported that early treatment in growing subjects with open-bite malocclusion showed scarce evidence of long-term success [18]. For this reason, the rationale of this study was to monitor treated and untreated patients with AOB, evaluate any modifications during their growth, and analyze any correlation with the expansion protocol.

This study aimed to evaluate the effects of a rapid maxillary expander with a crib on long-term changes in growing patients with anterior open bite. The null hypothesis was the absence of differences between the treated and non-treated groups. The specific aim of the study was to analyze skeletal and dental changes in vertical, transversal, and sagittal relationships evaluating growing patients with AOB and a maxillary contraction undergoing RME/c.

## 2. Materials and Methods

### 2.1. Study Design

The study was designed as a retrospective cohort study. The protocol and ethics followed the Declaration of Helsinki. The regional ethical review board (reference for the Magna Graecia University of Catanzaro, Catanzaro, Italy) approved the study (approval number: n.143/2018).

### 2.2. Study Sample

**Figure 1:**
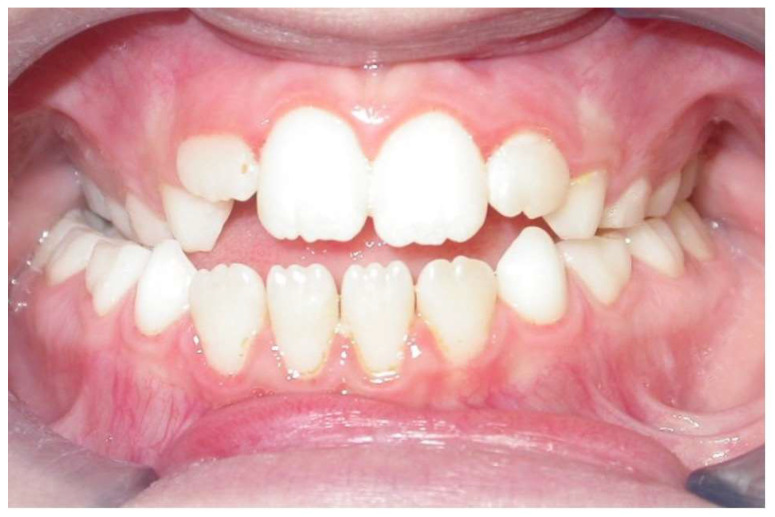
Intraoral frontal view of a patient with a negative overbite, transverse maxillary deficiency, and a complete eruption of upper incisors.

The study sample included the lateral cephalograms (LC) of patients aged 7 to 12 with anterior open-bite malocclusion who had undergone orthodontic treatment. The control group (CG), retrieved from the archives of the American Association of Orthodontists Foundation Craniofacial Growth Legacy Collection (http://www.aaoflegacycollection.org), included the LC of non-treated patients with anterior open bite. All the patients’ parents signed a specific informed consent form; then, the intraoral photographs and radiographic data were used for research purposes. The inclusion criteria were as follows: (1) negative overbite; (2) transverse maxillary deficiency with unilateral or bilateral cross-bite; (3) presence of first permanent molars; (4) complete eruption of the permanent central incisors (Figure 1); (5) no permanent teeth extraction before or during the treatment; (6) prepubertal stage of skeletal maturity according to the cervical vertebral maturation method (CS1 or CS2) [21]; (7) complete radiographic records and adequate follow-ups. Patients with facial malformations, syndromic diseases, and a history of previous jaw trauma were excluded.

### 2.3. Data Collection Method

**Figure 2:**
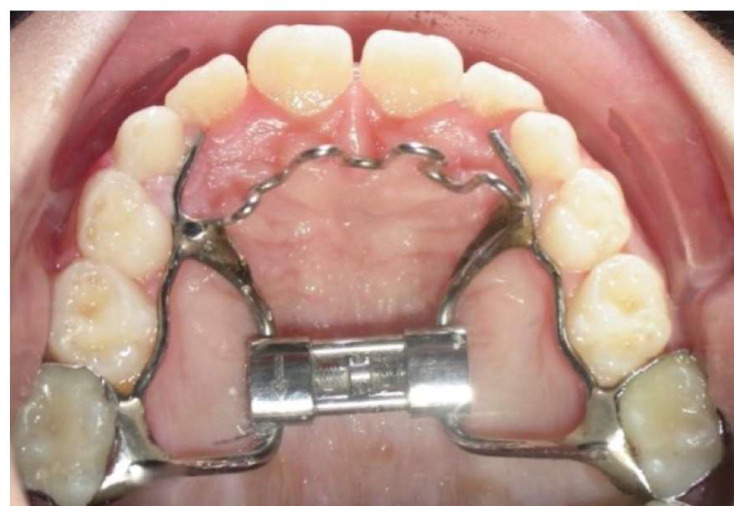
Occlusal view of a bonded rapid maxillary expander with a fixed palatal crib.

At baseline, the data collection method provided anamnestic and demographic data, radiographic tools, and intraoral photographs. The study group (TG) underwent a treatment protocol with RME/c (Figure 2). A two-banded rapid palatal expander (RPE) was bonded on the first permanent molars, and the expansion screw was activated twice a day for three weeks. After the active expansion, the device was left in place for eight months, stabilizing the conditions achieved during the screw activation (latency phase). Lateral cephalograms were recorded before treatment (T0), at the end of the latency phase (T1, approximately 12 months after T0 for CG), and before the fixed therapy (T2, approximately 5 years after T0 for CG), for both groups. Cephalometric analyses were performed using Delta Dent software (version 2.2.1 Outside Format, Spino d’Adda, Italy) by two expert investigators. All angular and linear cephalometric assessments analyzed are listed in Table 1 [22]. 

Vertical skeletal relationships were assessed by recording the values of mandibular divergence (SN^GoGn), maxillary divergence (SN^Ans-Pns), and maxillo-mandibular relationship (ANS-PNS^GoGn). Sagittal skeletal relationships were defined by the following angles: SNA, SNB, and ANB. Overjet and overbite values were also recorded for the dental relationships. 

### 2.4. Study Variables

The primary predictor variable was the therapeutic approach, distinguishing the TG from the CG.

The primary outcome variable focused on vertical skeletal and dental relationships (SN^GoGn, ANS-PNS^GoGn, and OVB).

The sagittal intermaxillary relationship and dentoskeletal features represented the secondary outcome variables.

Other study variables were recorded: the patients’ age and gender, their myofunctional problems, and changes between T0–T1, T1–T2, and T0–T2.

### 2.5. Statistical Analysis

A sample size calculation was performed, setting the following input values: 80% of power, 0.05 as level of significance, 1 as standard deviation, and 1 mm as difference in means to be recorded [16]. Each group would include 16 subjects.

The database was implemented using a dedicated Excel file (Microsoft, Redmond, WA, USA). The intra- and inter-rater agreement coefficients were calculated for the cephalometric measurements. The intra-rater reliability allowed the assessment of the agreement by the same investigator on the same cephalometric analysis repeated after 1 month. The inter-rater reliability allowed for the assessment of the agreement between the two investigators on the same analysis.

Descriptive statistics were calculated for all cephalometric measurements at T0, T1, and T2 in both groups. Mean and standard deviation or median and interquartile range were recorded for continuous quantitative variables in symmetric or asymmetric distributions, respectively. Absolute and relative frequencies were recorded for categorical data. To compare each outcome at different time points, bivariate analysis was performed using the paired Student *t*-test for normal distributions and the Wilcoxon test for asymmetrical distributions. A multivariate regression analysis was performed to evaluate the outcome variables in relation to the recorded measurements (the percentage of the R-squared coefficient was reported for each model). The level of significance was set at α = 0.05. A statistical analysis was performed using STATA software (STATA 11, StataCorp, College Station, TX, USA).

## 3. Results

### 3.1. Study Sample

The study sample included 32 patients, evenly divided into two study groups. All patients showed a dentoskeletal Class II malocclusion with a hyperdivergent pattern of growth (100%). The TG consisted of fourteen females (87.5%) and two males with a mean age of 7.5 ± 0.5 years at T0, while the CG included eleven females (68.7%) and five males with a mean age of 7.3 ± 0.7 years at T0. Most of the patients in both groups (62.5%) presented a bilateral cross-bite. At T0, all patients exhibited prepubertal skeletal maturity. Specifically, in the TG, 37.5% were classified as CS1, and 62.5% as CS2. In the CG, 62.5% were categorized as CS1, and 37.5% as CS2.

### 3.2. Comparative Analysis

The inter-rater agreement coefficient was κ = 0.91. The intra-rater agreement coefficient was κ = 0.93. At T0, all cephalometric variables showed no significant differences between the two study groups. All patients were affected by a Class II malocclusion with an increased vertical skeletal dimension, presenting an anterior open bite and a transversal skeletal cross-bite. By the end of the latency phase, all patients had corrected their maxillary contraction.

Bivariate statistical analysis was reported in Table 2. Furthermore, at T1, a significant decrease in jaw divergence was observed in the TG compared to the CG, according to the vertical skeletal relationship (*p* < 0.05). The intermaxillary angle was lower in the TG compared to the CG, without a statistically significant difference (*p* > 0.05). All patients showed an increased overbite, with a statistically significant difference between the TG and the CG (*p* < 0.05). Only patients in the TG recorded positive values for OVB.

At T2, statistical analysis recorded a significant decrease in maxillary and mandibular divergence in the TG compared to the CG (*p* < 0.05). Intermaxillary divergence showed a significantly reduced angle in the TG compared to the CG (*p* < 0.05). All treated patients maintained the open bite correction, showing higher OVB values compared to controls (*p* = 0.00000007). No patients showed relapse in terms of bad habits.

The multivariate regression analysis demonstrated a significant correlation between the vertical changes in the mandible (SN^GoGn angle) and the patients’ age: the value of the SN^GoGn angle decreases with age. A statistically significant correlation was found between the SN^GoGn angle and OVB values: at the long-term follow-up, SN^GoGn angle values decreased with increasing post-treatment OVB values. This model explained 64% of the variability (*p* < 0.0001).

## 4. Discussion

This retrospective study aimed to assess both skeletal and dental changes in vertical and sagittal relationships by comparing growing patients with AOB who had undergone RME/c treatment with a control group of untreated subjects of the same age. As reported by Lin et al., open-bite malocclusion can be related to both skeletal and dentoalveolar alterations in the maxilla and the mandible [2]. Hyperdivergency can be a genetic risk factor of AOB due to the vertical growth pattern of the jaw [23,24,25,26,27]. A mandibular post-rotation can be a direct consequence of the unbalanced vertical growth between the molar region and the condylar area [23,24,25,26,27,28]. Furthermore, an open bite is often associated with decreased transversal maxillary development and, frequently, with the presence of a posterior cross-bite and consequent atypical swallowing [29]. In this case, orthodontic therapy should aim to correct skeletal, dentoalveolar, and functional alterations as the primary outcome. Maxillary expansion and correction of bad habits are fundamental in the early treatment of open bite malocclusion.

To define any dental or skeletal changes after maxillary expansion, all patients were evaluated at a short-term follow-up (end of the active expansion phase, approximately 12 months after T0) and a long-term follow-up (before the fixed orthodontic therapy, approximately 5 years after T0), comparing the results with the initial records. Two homogeneous study groups were selected, corresponding to vertical, transversal, and sagittal skeletal and dental characteristics to increase the reliability of each outcome.

The results showed both skeletal and dental improvements after orthodontic therapy. At the end of the latency phase (T1), all patients in the TG showed a positive OVB, achieving the primary treatment goal of an anterior seal. The mean increase in overbite was 4.4 mm after maxillary expansion therapy. These results align with those of Mucedero et al., who reported long-term outcomes in patients with anterior open bite treated by a quad-helix appliance in addition to the palatal crib [28]. They found an OVB increase of 4.2 mm in the treated group, with a significant improvement compared to the control group [28]. At a short-term follow-up, Torres et al. reported a mean overbite improvement of 3.86 mm, while Pedrin and colleagues achieved a greater average value of 5.01 mm with their protocol of palatal crib in addition to a high-pull chin cup [30,31]. In this study, a significant difference was recorded at both T1 and T2 when comparing the improvement in overbite between the TG and the CG with no post-treatment relapse, thus demonstrating the efficacy of the therapeutic approach. Dental changes included lingual tipping of upper and lower incisors at both short- and long-term follow-ups, contributing to open bite closure. According to the literature, this therapeutic outcome could derive from myofunctional rehabilitation, with the interruption of tongue thrusting and non-nutritive sucking habits promoted by using a palatal crib [32,33]. Although molar extrusion with mandibular post-rotation could be a side effect of a rapid maxillary expander, in this study it may have been balanced by eliminating oral habits and restoring myofunctionality, as reported by Mousa and colleagues [33,34].

Transversal correction also induced skeletal modifications of the maxillomandibular relationships with significant differences between treated and untreated patients. On the sagittal plane, both the TG and the CG showed a limited improvement of maxillary and mandibular position, recording a decrease in ANB° of less than 1°. The statistical analysis confirmed the previous results by other authors who evaluated the long-term outcomes of open-bite patients treated with maxillary expansion and bite-blocks [16]. In accordance with their conclusions, no statistically significant difference in sagittal skeletal changes was highlighted between the study groups at different time points.

In vertical skeletal analysis, no evident modifications were recorded on the maxillary relationship with respect to the cranial base (approximately 1.0°). This value is slightly lower compared to previous studies reporting 1.2° and 1.8° of downward rotation of the palatal plane [28,35]. In contrast, the TG exhibited a notable reduction in the maxillo-mandibular angle by an average of 2.0° at both T1 and T2. These findings highlight a distinct pattern in the TG, showing a smaller maxillo-mandibular angle over the observation period. This outcome is in line with the findings of Mucedero et al., who found a significant difference in the same entity at the short-term follow-up [28]. According to the literature, patients in the TG exhibited a reduction of mandibular divergence and gonial angle [28]. Maxillary expansion determined, on average, about 2.0° of mandibular counterclockwise rotation, contributing significantly to the open bite closure. These results are slightly higher than those of other authors, who recorded a mean value ranging between 1.1 and 1.4 mm when evaluating the SN-GoMe angle in open-bite patients treated with removable appliances [33,36,37]. It is probable that the different designs and the discontinuity of use can influence the skeletal effects.

In this study, a fixed two-band maxillary expander with a tongue crib was employed for rapid maxillary expansion, transmitting substantial forces directly to the maxilla through the anchored teeth [38,39]. Initially, this process induces hyalinization of the periodontal ligament in the first period, preventing tooth movement and achieving an orthopedic effect [38]. Further on, this protocol can yield more favorable skeletal changes when performed before peak pubertal growth, as evidenced in this study [29,39]. It is important to note, however, that discomfort associated with the separation of the palatine suture could potentially occur, leading to pain; nevertheless, this is an exceptionally rare phenomenon [38,39]. According to the literature, transversal correction influenced the maxillomandibular vertical and sagittal relationships, achieving a long-term improvement in overbite [33]. Specifically, after therapy, significant changes allowed the open bite closure, including the anterior rotation of the mandible, the decrease of mandibular divergence, and the reduction of gonial angle as skeletal factors. On the other hand, the reduction of upper incisor inclination, the increase of lingual lower incisor tipping, and the reduction of the antero-posterior position of upper and lower incisors were the most effective dentoalveolar modifications. As reported by Giuntini, Torres and Teixeira, tongue crib increases the tone of the labial muscles, promoting a backward pressure on mandibular incisors, allowing normalization of myofunctional activity, and eliminating tongue thrusting or non-nutritive sucking habits [30,40,41].

While various authors have examined the outcomes of diverse therapeutic approaches to anterior open bite malocclusion, to the best of our knowledge, this study marks a pioneering investigation into the short- and long-term effects following treatment with a palatal expander with a tongue crib. Notably, it includes a comparative analysis with an untreated control group, further enhancing the innovative nature of this research. In terms of limitations, despite the control group (CG) patients exhibiting the same initial characteristics as the treatment group (TG), they were recruited from a historical database due to ethical constraints, given the impracticality of avoiding therapeutic intervention in open bite cases in the present context. This could introduce potential biases related to variations in treatment approaches over time, potentially limiting the generalizability of these findings. Furthermore, while the sample size calculation established a necessity of 16 patients in each group, the findings could benefit from larger cohorts. Finally, a precise evaluation of molar extrusion after maxillary expansion was not recorded, although growth vertical control could be deduced by the improvement of facial divergency at both short- and long-term follow-up.

## 5. Conclusions

In conclusion, this study presents a comprehensive exploration of the short- and long-term effects of treating anterior open-bite malocclusion with a palatal expander featuring a lingual crib (RME/c). The null hypothesis was rejected based on the observed differences between the treated and non-treated groups. The application of maxillary expansion combined with a tongue crib proved to be an effective treatment strategy for growing patients with an open bite and poor oral habits. These findings demonstrated significant and stable dental and skeletal changes at both short-term and long-term follow-ups. Notably, the treatment led to overbite correction, controlled vertical growth, and successful myofunctional rehabilitation.

## Figures and Tables

**Table 1:** List of angular and linear cephalometric assessments.

Cephalometric Measurement (Unit of Measurement)	Calculation Method	Abbreviation
Sagittal analysis		
Position of the maxilla (°)	Angle: sella–nasion–point A	SNA
Position of the mandible (°)	Angle: sella–nasion–point B	SNB
Intermaxillary relationship (°)	Angle: point A–nasion–point B	ANB
Vertical analysis		
Vertical intermaxillary relationship (°)	Angle between palatal plane and mandibular plane	AnsPns.GoGn
Divergence of the maxilla (°)	Angle between sella–nasion plane and palatal plane	SN.AnsPns
Divergence of the mandible (°)	Angle between sella–nasion plane and mandibular plane	SN.GoGn
Gonial angle (°)	Angle: condylion–gonion–gnathion	CoGoMe
Dentobasal analysis		
Inclination of the lower incisors (°)	Angle between lower incisor axis and mandibular plane	L1.GoGn
Inclination of the upper incisors (°)	Angle between upper incisor axis and palatal plane	U1.AnsPns
Anteroposterior position of the lower incisor (mm)	Distance between pogonion and the projection of the lower incisor axis perpendicular to the Frankfurt plane	L1-Pg
Anteroposterior position of the upper incisor (mm)	Distance between anterior nasal spine and the projection of the upper incisor axis perpendicular to the Frankfurt plane	U1
Molar relationship (mm)	Relationship between upper and lower first molar	U6^L6
Overbite (mm)	Vertical distance between the margins of upper and lower incisors	OVB
Overjet (mm)	Horizontal distance between the margins of upper and lower incisors	OVJ

(°) = degrees; (mm) = millimeters; palatal plane = plane passing through the anterior nasal spine (Ans) and the posterior nasal spine (Pns); mandibular plane = plane passing through the gonion (Go) and gnathion (Gn) points; lower incisor axis = plane passing through the apical point of the inferior incisor (Ap1l) and the incisal point of the inferior incisor (In1l); upper incisor axis = plane passing through the apical point of the upper incisor (Ap1u) and the incisal point of the upper incisor (In1u) and palatal plane.

**Table 2:** Bivariate comparative analysis between the treatment group and the control group at different time points.

	T0		*p*-Value	T1		*p*-Value	T1-T0		*p*-Value	T2		*p*-Value	T2-T1		*p*-Value	T2-T0		*p*-Value
	TG	CG		TG	CG		TG	CG		TG	CG		TG	CG		TG	CG	
Sagittal analysis																		
SNA	82.6 ± 3.4	83.9 ± 5.8	NS	82.6 ± 2.9	84.5 ± 6.1	NS	0.09 ± 1.80	0.57 ± 2.30	NS	83.1 ± 3.2	84.3 ± 5.3	NS	0.49 ± 1.17	−0.17 ± 3.18	NS	0.58 ± 2.88	0.40 ± 3.83	NS
SNB	78.2 ± 2.1	78.8 ± 5.3	NS	79.1 ± 2.3	80.4 ± 5.3	NS	0.99 ± 1.55	1.62 ± 1.96	NS	79.6 ± 2.3	80.7 ± 5.5	NS	0.47 ± 1.35	0.27 ± 2.85	NS	1.46 ± 1.91	1.89 ± 3.84	NS
ANB	4.6 ± 2.9	5.01 ± 2.3	NS	3.72 ± 1.7	4.16 ± 1.6	NS	−0.88 ± 1.45	−0.84 ± 1.75	NS	4.03 ± 1.9	3.9 ± 1.5	NS	0.31 ± 0.77	−0.26 ± 1.80	NS	−0.56 ± 1.25	−1.11 ± 1.98	NS
Vertical analysis																		
AnsPns.GoGn	28.3 ± 3.2	27.2 ± 4.9	NS	26.7 ± 3.3	28.9 ± 4.6	NS	−1.62 ± 0.52	1.71 ± 1.80	*	25.2 ± 3.1	30.2 ± 4.5	*	−1.46 ± 0.43	1.29 ± 0.76	*	−3.08 ± 0.63	2.99 ± 2.22	*
SN.AnsPns	6.6 ± 2.1	7.3 ± 3.2	NS	6.1 ± 0.8	8.2 ± 2.9	*	−0.51 ± 1.84	0.83 ± 0.74	*	5.5 ± 1.7	9.2 ± 2.8	*	−0.62 ± 1.46	1.05 ± 1.14	*	−1.14 ± 2.82	1.88 ± 1.45	*
SN.GoGn	34.6 ± 4.9	34.8 ± 6.2	NS	32.6 ± 5.1	35.3 ± 5.9	*	−1.93 ± 1.39	0.52 ± 0.98	*	29.9 ± 4.5	36.1 ± 5.6	*	−2.69 ± 2.99	0.79 ± 1.31	*	−4.63 ± 3.49	0.88 ± 1.93	*
CoGoMe	128.1 ± 4.7	126.7 ± 6.9	NS	126.6 ± 3.5	126.4 ± 5.8	NS	−1.45 ± 1.50	−0.37 ± 3.61	NS	124.4 ± 2.6	127.4 ± 5.6	NS	−2.20 ± 2.15	1.07 ± 1.72	*	−3.65 ± 3.01	0.71 ± 4.82	NS
Dentobasal analysis																		
L1.GoGn	100.2 ± 7.7	97.3 ± 7.7	NS	99.5 ± 5.9	97.7 ± 5.8	NS	−0.69 ± 3.09	0.44 ± 5.22	NS	97.8 ± 5.8	97.2 ± 4.9	NS	−1.63 ± 5.85	−0.49 ± 4.93	NS	−2.33 ± 8.88	−0.06 ± 7.06	NS
U1.AnsPns	115.3 ± 7.0	117.4 ± 7.7	NS	114.4 ± 6.6	117.4 ± 6.4	NS	−0.89 ± 7.24	0.06 ± 6.75	NS	109.9 ± 7.0	116.4 ± 5.9	*	−4.50 ± 5.92	−1.03 ± 5.67	NS	−5.39 ± 11.24	−0.98 ± 6.61	NS
L1	2.8 ± 3.5	5.4 ± 2.8	NS	3 ± 2.3	5.6 ± 2.8	*	0.17 ± 1.49	0.24 ± 0.89	NS	2.9 ± 1.8	5.7 ± 2.6	*	−0.14 ± 0.73	0.11 ± 0.77	NS	0.03 ± 2.03	0.34 ± 1.34	NS
U1	0.4 ± 2.3	−2.6 ± 2.1	NS	1 ± 1.0	−2.2 ± 2.0	*	0.61 ± 1.58	0.44 ± 1.28	NS	1.6 ± 1.1	−1.1 ± 2.4	*	0.54 ± 1.00	1.15 ± 1.88	NS	1.15 ± 2.40	1.59 ± 2.22	NS
U6^L6	0.7 ± 0.2	0.8 ± 0.2	NS	0.7 ± 0.2	0.9 ± 0.1	*	0.02 ± 0.10	0.08 ± 0.19	NS	0.8 ± 0.2	1 ± 0.1	*	0.07 ± 0.10	0.16 ± 0.13	*	0.10 ± 0.13	0.23 ± 0.20	*
OVB	−2.9 ± 1.1	−4.4 ± 1.9	NS	1.5 ± 0.6	−3.3 ± 1.3	*	4.49 ± 0.92	1.04 ± 1.13	*	1.8 ± 0.6	−2.1 ± 1.3	*	0.32 ± 0.20	0.32 ± 0.20	*	4.81 ± 0.96	2.30 ± 1.65	*
OVJ	2.7 ± 1.3	2.6 ± 2.8	NS	2.6 ± 1.0	3 ± 2.3	NS	−0.06 ± 0.43	0.43 (1.02)	NS	2.6 ± 0.7	3.7 ± 2.1	NS	0.00 ± 0.62	0.65 ± 0.58	*	-0.06 ± 0.98	1.08 ± 1.48	*-

NS = not significant *p*-value; * = significant *p*-value; SNA = angle: sella—nasion—point A; SNB = angle: sella—nasion—point B; ANB = angle: point A—nasion—point B; AnsPns.GoGn = angle between palatal plane and mandibular plane; SN.AnsPns = angle between sella—nasion plane and palatal plane; SN.GoGn = angle between sella—nasion plane and mandibular plane; CoGoMe = angle: condylion—gonion—gnathion; L1.GoGn = angle between lower incisor axis and mandibular plane; U1.AnsPns = angle between upper incisor axis; L1 = distance between pogonion and the projection of the lower incisor axis perpendicular to the Frankfurt plane; U1 = distance between anterior nasal spine and the projection of the upper incisor axis perpendicular to the Frankfurt plane; U6^L6 = relationship between upper and lower first molar; OVJ = overjet; OVB = overbite.

## Data Availability

Data will be available from corresponding authors.
